# Gene Expression Profile (GEP) Comparison of Atypical Fibroxanthoma (AFX) and Pleomorphic Dermal Sarcoma (PDS)

**DOI:** 10.3390/cancers18060934

**Published:** 2026-03-13

**Authors:** Alessio Giubellino, Gerardo Cazzato, Mario Della Mura, Giuseppe Broggi, Alessandro Rizzo, Nehaaluddin Azmi, Carlos A. Torres-Cabala, Sarah Munro, Faqian Li

**Affiliations:** 1 Department of Laboratory Medicine and Pathology, University of Minnesota, Minneapolis, MN 55455, USA; 2 Masonic Cancer Center, University of Minnesota, Minneapolis, MN 55455, USA; 3 Section of Molecular Pathology, Department of Precision and Regenerative Medicine and Ionian Area (DiMePRe-J), University of Bari Aldo Moro, 70121 Bari, Italy; 4 Department of Medical and Surgical Sciences and Advanced Technologies “G.F. Ingrassia”, Anatomic Pathology, University of Catania, 95131 Catania, Italy; giuseppe.broggi@gmail.com; 5 IRCCS Istituto Tumori “Giovanni Paolo II”, 70124 Bari, Italy; 6 Department of Pathology and Laboratory Medicine, University of Texas MD Anderson Cancer Center, Houston, TX 77030, USA; 7 Minnesota Supercomputing Institute, University of Minnesota, Minneapolis, MN 55455, USA; 8 Department of Pathology and Laboratory Medicine, University of Texas San Antonio, San Antonio, TX 78249, USA

**Keywords:** atypical fibroxanthoma, pleomorphic dermal sarcoma, cutaneous undifferentiated pleomorphic sarcoma, RNA, transcriptome

## Abstract

**Background/Objectives**: Atypical fibroxanthoma (AFX) and cutaneous undifferentiated pleomorphic sarcoma (cUPS)/pleomorphic dermal sarcoma (PDS) are related dermal neoplasms of uncertain histogenesis that occupy opposite ends of a shared clinical and histopathologic spectrum, with AFX displaying typically low-grade behavior and PDS representing its more aggressive counterpart. The recent literature has confirmed that AFX and PDS also overlap at the molecular and genomic levels; however, little is known about their gene-expression profiles. **Methods**: We performed gene-expression profiling using RNA sequencing with a Pan-Cancer RNA Panel on a small series of AFX and PDS samples. **Results**: Unsupervised cluster analysis showed a clear separation between the two groups. We confirmed a TP53 UV-radiation signature in both. However, while AFX and PDS share common DNA mutation profiles in our cohort, RNA sequencing reveals distinct gene-expression signatures that may aid in differentiating these related tumors. In particular, the MAPK pathway, cell adhesion, DNA repair, EMT-like signatures and inflammatory responses play key roles in distinguishing the two groups, at least in our limited cohort, consistent with their differing biological behavior. Differences in the expression of receptor tyrosine kinases were also observed. **Conclusions**: Gene-expression profiling have the potential to be a valuable tool for distinguishing AFX from PDS, clarifying their positions at opposite ends of a spectrum and providing deeper insight into the biology of these neoplasms.

## Simple Summary

Atypical fibroxanthoma (AFX) is a low-grade tumor related to the more aggressive cutaneous undifferentiated pleomorphic sarcoma (cUPS)/pleomorphic dermal sarcoma (PDS). While they both share a common DNA profile, we explore their RNA expression profile and show that it is different, including specific MAPK pathway, DNA repair and inflammatory response profiles, at least in our cohort. This different gene expression profile can be potentially leveraged to improve correct classification and potentially to define future potential targets for therapy.

## 1. Introduction

Atypical fibroxanthoma (AFX) and cutaneous undifferentiated pleomorphic sarcoma (cUPS)/pleomorphic dermal sarcoma (PDS) are dermal tumors that exist along a clinical and histopathologic spectrum [1].

Although their histogenesis remains debated, both typically arise on sun-damaged skin, implicating UV radiation in their etiology [2]. They most commonly occur in older adults, particularly men, and present as rapidly growing nodules, which may sometimes lead to ulceration of the overlying epidermis. Their clinical and histologic features can mimic other cutaneous malignancies, such as spindle cell squamous cell carcinoma or melanoma. Therefore, biopsy is essential for definitive diagnosis and should be complemented by an appropriate immunohistochemical panel to distinguish them from other cutaneous tumors [3]. Histologically, both AFX and PDS are highly cellular and composed of pleomorphic cells with nuclear enlargement and atypia, as well as frequent mitotic figures, some of which may be atypical [4].

Although these tumors may appear similar cytologically, careful histopathologic examination of a specimen encompassing the entire lesion can distinguish one from the other. Key features favoring a diagnosis of PDS include invasion of the subcutaneous adipose tissue, tumor necrosis, and vascular or perineural invasion [4], all features portending a more aggressive lesion. Immunohistochemistry is essential for ruling out tumor mimickers—such as squamous cell carcinoma, melanoma, leiomyosarcoma, and angiosarcoma (WHO, 2025)—but it provides no meaningful help in distinguishing AFX from PDS, as neither lesion expresses specific markers that allow for a definitive separation.

From a genetic point of view, both tumors appear to have a similar background. For example, *TP53* is mutated in both AFX and PDS [2], likely as part of UV damage signature. In addition, *FAT1*, *NOTCH1*/2, *CDKN2A*, and *TERT* promoter mutations have been recurrently described in both lesions [5]. Other studies have also revealed that both AFX and PDS have a high tumor mutational burden (TMB) [6]. More recently, limited gene expression profiles have been reported [7,8,9]. However, direct comparison of gene expression of AFX with PDS in bulk RNA is currently lacking.

For both AFX and PDS, complete surgical excision is the treatment of choice [10]. In particular, for AFX, Mohs micrographic surgery (MMS) is considered the gold standard with a very good outcome and very low recurrence rates [11,12,13]. For PDS, wide local excision (WLE) is instead often necessary. In rare cases, radiation therapy can be adjunctively employed to ensure complete eradication [10,14]. When completely removed, AFX behaves in a benign fashion, with virtually no risk of recurrence or metastatic spread [13]. By contrast, local recurrence occurs in 24% of PDS cases and distant metastasis in 12% [15], making regular follow-up essential.

In our study, we present the RNA expression profiles of a selected gene panel in nine AFX cases compared with three PDS cases, highlighting the differences in gene expression to investigate whether there are biological distinctions in the transcriptomic profiles of these two genetically and cytologically similar neoplasms, which, however, differ in their aggressiveness.

## 2. Materials and Methods

### 2.1. Patients Selection

Formalin-fixed, paraffin-embedded (FFPE) samples of both AFX and PDS were obtained from our surgical pathology service as excisional biopsies. Clinical information was retrieved from our centralized medical record system. The study was approved by the University of Minnesota Institutional Review Board (IRB) (IRB # 00022007) and conducted in accordance with the Declaration of Helsinki.

### 2.2. RNA Sequencing

Unstained slides were macro-dissected to collect the tumor. Total RNA was extracted and purified using a RNeasy FFPE Kit (Qiagen, Germantown, MD, USA). RNA samples were then quantified and analyzed for quality (Agilent RNA 6000 Nano Kit; Agilent Technologies, Santa Clara, CA, USA). Library preparation and targeted gene enrichment was performed with the TruSight RNA Pan-Cancer Panel Kit, targeting 1385 cancer-related genes, according to manufacturer’s protocol (Illumina, San Diego, CA, USA). Libraries were sequenced on the Illumina NextSeq 550 System (Illumina, San Diego, CA, USA).

### 2.3. Bioinformatics Analysis

FASTQ file analysis was performed using the Illumina BaseSpace RNA-Seq Alignment Application 2.0.2 as described previously [16].

### 2.4. RNA Variant Analysis

Variant files were annotated with clinical genomic information including gnomAD minor allele fractions, COSMIC cancer listings, and NCBI ClinVar clinical significance. Then, they were quality filtered and reviewed by a board-certified molecular pathologist as described previously [16].

### 2.5. Differential Gene Expression and Pathway Analysis

Gene-level counts from the BaseSpace RNA-Seq Alignment Application were analyzed using custom R scripts and open-source R packages ( version 4.5.1). We analyzed 12 patients, nine patients presenting with AFX (AFX 1–9) and 3 patients presenting with PDS (PDS 1–3). We removed any genes that did not have at least 1 cpm (count per million) in at least 2 samples. We used the quasilikelihood test in edgeR (version 4) [17] for differential expression analysis between the AFX and PDS samples. The threshold for significance was based on an FDR = 0.05. We used clusterProfiler (version 4.15.2) [18] for the following two types of pathway analysis: GO term overrepresentation analysis and gene set enrichment analysis (GSEA) [19] with the following MSigDb collections: Hallmark, C2 (Reactome subset only), C5 (GO BP and MF subsets only), and C6 (Oncogenic signatures) [20]. A useful comparison of normalization strategies and differential expression approaches in RNA-seq datasets can also be found in Lin et al. [21]. For overrepresentation analysis we tested the top positive and negative DE genes separately. For GSEA the ranking metric was the negative log10 *p*-value multiplied by the sign of the fold change.

## 3. Results

### 3.1. Clinical and Histopathologic Features

A total of 12 tumors were included in the study, comprising nine AFX and three PDS cases. Table 1 summarizes the clinical and histopathologic characteristics of the samples in our cohort.

**Figure 1:**
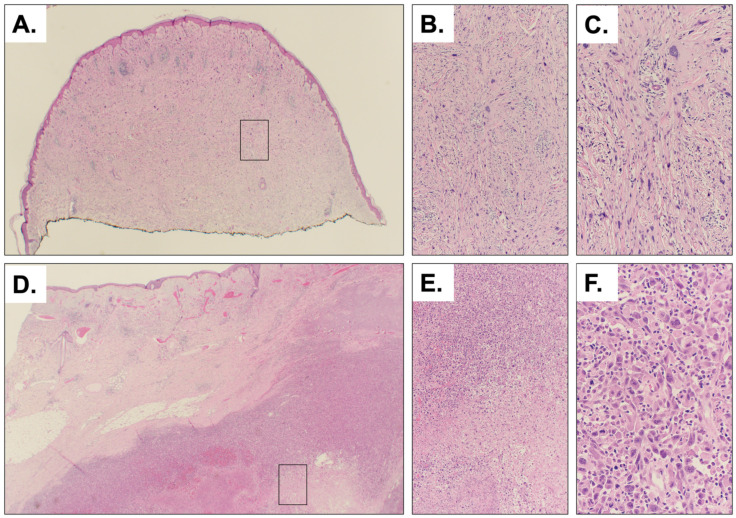
Histology of a representative AFX and PDS. Low power view of a typical example of AFX from our cohort showing a dermal-based tumor ((**A**), 2× magnification; inset highlights section magnified in panel (**B**)). At lower power we appreciate irregularly arranged spindle and epithelioid cells in the dermis ((**B**), 10× magnification). At 20× magnification it is possible to see the pleomorphism of the cells within fascicles (**C**). Low power view of a typical example of PDS showing involvement of the deep dermis and subcutaneous tissue ((**D**), 2× magnification; inset highlights section magnified in panel (**E**)). In this example mostly epithelioid cells are seen adjacent to tissue necrosis ((**E**), 10× magnification). At higher power we can appreciate pleomorphism and atypical cells with occasional nucleoli and mitoses ((**F**), 20× magnification).

AFXs were more common in men, as expected, with a mean age at diagnosis of 79 years (range 65–91), and the scalp was the most frequent site; only two cases presented as ulcerated lesions. No recurrences were observed following MMS. All PDS cases occurred in men, with a mean age at diagnosis of 55 years (range 42–69). All three tumors were ulcerated, invaded the subcutaneous tissue (with two cases also involving deeper layers), and exhibited necrosis and high mitotic counts. Two patients died from the disease. Figure 1 illustrates a typical case of AFX (Figure 1A–C) and PDS (Figure 1D–F).

### 3.2. Mutational Profile

**Figure 2:**
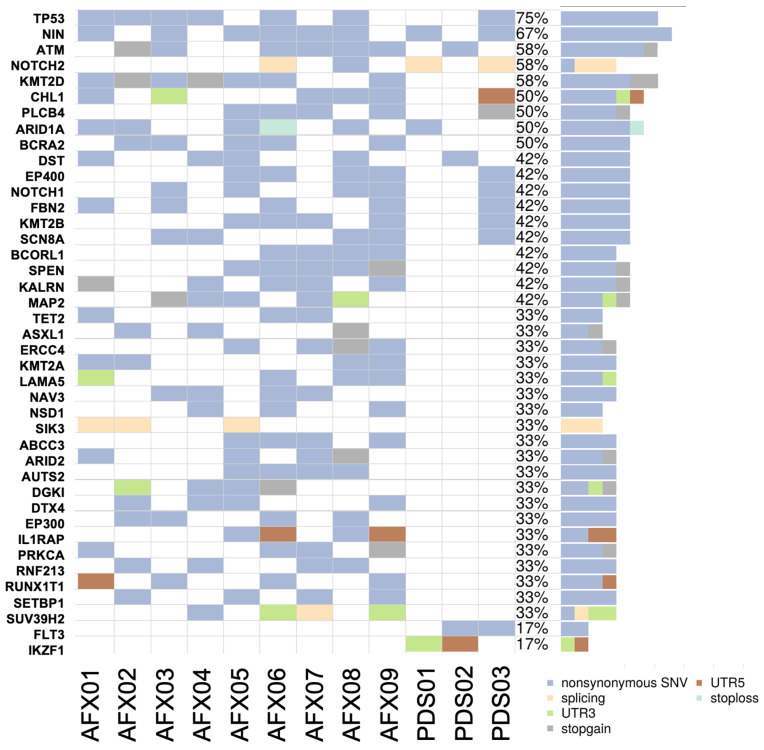
Gene mutational profiles.

Analysis of the sequencing data revealed a heterogeneous mutational landscape across the samples in our study (Figure 2). The most frequently mutated genes included *TP53* (75% of the samples), *NIN* (67%), *ATM* (58%), *NOTCH2* (58%), and *KMT2D* (58%). Several other genes were also mutated, with AFX cases exhibiting a higher overall mutation burden than PDS cases within the limits of our gene panel. Genes uniquely mutated in AFX included *KMT2D*, *BRCA2*, *KMT2A*, *ARID2*, *PRKCA*, and *RUNX1T1*, whereas only a few genes were uniquely mutated in PDS, including *FLT3*. No gene fusions were detected in any of the samples.

### 3.3. Gene Expression and Pathway Analysis Profiles

**Figure 3:**
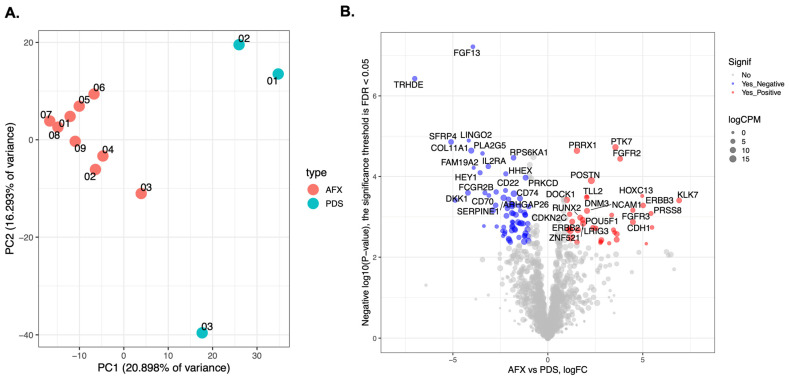
(**A**) Principal component analysis (PCA). PCA shows clear clustering of PDS and AFX tumors based on gene expression profiles (PC1 vs. PC2). (**B**) Volcano plot of the differentially expressed genes between AFX and PDS.

We analyzed gene expression profiles from the RNA-seq data. Expression data for AFX and PDS were first visualized using principal component analysis (PCA), with PC1 and PC2 representing the first and second principal components, respectively (Figure 3A).

We then analyzed the differential gene expression between AFX and PDS cases and presented the results in a volcano plot in Figure 3B and Supplemental Table S1.

Among the genes upregulated in AFX were PTK7, *FGFR2*, ERBB2, *ERBB3*, DOCK1 and *WNT3*, while downregulated genes include *IL2RA*, *JAK3*, *CXCR4*, *PLA2G5*, *FOS*, *RAC2*, *FLI1*, *VEGFA* and *FGF13*. In PDS we found upregulation of *CXCR4*, DKK1, PTK2B, WNT7B, NGFR, MAP3K1, COL9A3 and *COL11A1* as well as downregulation of *NCAM1*, *PAK6*, *CDKN2C*, *FGFR3* and *RET*. We also compared the genes with differential gene expression in our samples with a list of about 50 genes reported in the literature as either mutated or differential expressed in AFX and PDS (Supplemental Table S2) and we found an overlap in few genes, including NOTCH 1, CD22 and CD74 which were downregulated in our PDS samples and CDH1 which was downregulated in our AFX samples (Supplemental Figure S1).

For pathway analysis, we first created a ranked gene list for each group. Genes that were differentially expressed between AFX and PDS (FDR ≤ 0.01) were then subjected to over-representation analysis for Gene Ontology (GO) terms. As shown in Supplemental Table S3, PDS samples were enriched for GO terms associated with the MAPK pathway, cell adhesion, positive regulation of signal transduction and immune system regulation.

**Figure 4:**
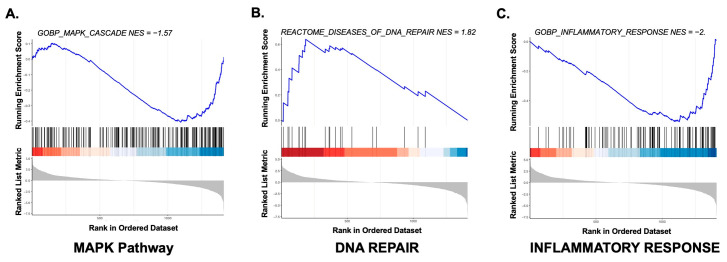
GSEA running enrichment score plots for (**A**) MAPK PATHWAY, (**B**) DNA REPAIR and (**C**) INFLAMMATORY RESPONSE.

We then performed gene set enrichment analysis (GSEA) on the same set of differentially expressed genes (Supplemental Table S4). As shown in Figure 4, enrichment plots for MAPK pathway, DNA repair and inflammatory response-related genes demonstrate a clear separation between AFX and PDS cases. Supplemental Figure S2 shows the relative heatmaps. In particular, DNA repair was predominantly upregulated in AFX specimens, whereas MAPK pathway and inflammation-related genes were upregulated in PDS cases.

## 4. Discussion

The current consensus is that AFX and PDS represent opposite ends of a spectrum of lesions, with PDS representing the more aggressive form of AFX. Some tumors clearly fall at one end of this spectrum, while others may represent intermediate lesions. This highlights the importance of a thorough and complete evaluation of these lesions, sometimes deferring a final diagnosis until re-excision in cases of superficial sampling. Some authors argue that any of these tumors, even with only superficial or minimal subcutaneous involvement, should be diagnosed as PDS and treated more aggressively to prevent recurrence [22]. In contrast, others prefer to reserve the PDS designation for cases in which the entire lesion is visualized and exhibits all features of malignancy, including deep extension, necrosis, and vascular or perineural invasion.

Currently, the distinction between these entities remains challenging. A comprehensive immunohistochemical panel is employed to rule out other tumors, including high-grade neoplasms such as spindle cell melanoma (S100, SOX10), sarcomatoid carcinoma (cytokeratins, p63/p40), leiomyosarcoma (desmin, caldesmon) and angiosarcoma (CD31, ERG). Some authors have suggested specific immunohistochemical algorithms [3], but the dependence on negative markers, without real positive lineage-specific identifiers, is a source of uncertainty. This is particularly problematic for “borderline” cases. There are even rare reports where IHC may even be misleading in the interpretation of these tumors; for example, Helbig et al. [23] illustrated cases where these tumors stained positive for some melanoma markers, and only through genomic analysis was the pathologist able to realize the true nature of the tumor. Thus, as it is true for many other lesions, we can never rely only on a single marker for an accurate diagnosis, but we need to rely on multiple sources of evidence for a more definitive interpretation.

In our small study, we aimed to move beyond morphology and examine the gene expression profiles underlying these entities, identifying several important differences between AFX and PDX that may help justify the differences in their biological behavior.

Our comparative transcriptomic profile between AFX and PDS may help to generate potential hypotheses and molecular explanations for the differences in invasiveness and clinical behavior observed in these neoplasms. For example, our findings may suggest that PDS is characterized by a molecular transformation that exchanges mesenchymal lineage stability for an aggressive and pro-invasive phenotype.

The expression patterns of genes involved in cellular identity and extracellular matrix (ECM) remodeling may provide a clue to the differences between the two entities. AFX cases showed upregulation of PRRX1 and CDH11. PRRX1 is a regulator of the myofibroblastic lineage [24] and its relative preservation in AFX may suggest a tumor that remains biologically anchored to its mesenchymal identity. In contrast, PDS samples in our cohort exhibit a downregulation of these lineage markers alongside an upregulation of genes that appear related to an invasive machinery. In particular, the upregulation of CXCR4 and PTK2B (FAK2) in PDS may highlight a potential mechanism for directed migration and invasion. The CXCR4-CXCL12 axis is a well-established driver of site-specific infiltration in various malignancies [25]. This migratory capacity is reinforced by structural remodeling, as evidenced by the upregulation of COL11A1 and COL9A3 in PDS. COL11A1 has been identified as an important marker for the transition from a localized to an invasive phenotype in both carcinomas and sarcomas [26,27], facilitating the deep infiltration that defines PDS.

Moreover, our data may also suggest a shift in growth signaling dependencies. AFX demonstrates higher levels of some receptor tyrosine kinases (RTKs) such as FGFR2, ERBB2, and PDGFRA. This data may suggest that AFX in our cohort may rely on growth factor-dependent pathways. On the other hand, PDS appears to bypass these constraints through unregulated MAPK signaling and the upregulation of IGF1 and MAP3K1. The induction of IGF1 in this context may establish an autocrine mitogenic loop that drives rapid proliferation and survival in the deep dermal environment. This shift is further emphasized by the loss of tumor suppression, specifically the downregulation of NOTCH1 and CDKN2C (p18) in PDS. As NOTCH1 is a recognized tumor suppression gene in some cutaneous tumors [28,29,30], its suppression in PDS may permit a more aggressive and dedifferentiated state needed for tumor progression.

Our analysis also revealed a distinct and complex inflammatory component within the PDS microenvironment. GSEA confirmed enrichment for inflammatory responses, characterized by the upregulation of IL2RA (CD25), JAK3, and PRF1 (Perforin 1). While all PDS cases in our cohort were ulcerated (and only two in the AFX cases)—a factor that typically introduces acute inflammatory and potential pathogens from the environment—making it difficult to entirely exclude a non-tumoral immune response, our data may suggests a *bona fide* anti-tumor immune signature in our samples rather than simple superinfection. The absence of significant upregulation in acute markers such as S100A8/A9 and TLR2/4 argues against a purely bacterial-driven response. Instead, the high expression of PRF1 and IL2RA may be a clue to a “hot” tumor microenvironment rich in cytotoxic and regulatory T-cells [31,32]. However, the concomitant downregulation of CD74 and CD22 may suggest a sophisticated immune-evasion strategy, where PDS established a proinflammatory but immunologically suppressed niche to support its growth.

Furthermore, PDS appears to utilize EMT-like transcriptomic programs—such as the downregulation of CDH1 and the upregulation of CXCR4—to achieve its higher degree of motility and invasiveness [33]. The preservation of DNA repair pathways and adhesion molecules like ITGAV in AFX may be an explanation for its more indolent growth pattern, whereas the loss of these restraints in PDS may mark the transition to a more aggressive behavior. Additional studies on a larger cohort may further confirm our findings and clarify if these specific pathways could be targeted therapeutically, perhaps through the inhibition of the CXCR4 or MAPK axes in advanced PDS.

We need to stress that, given the small number of samples, our data are exploratory in nature, and the above discussion should be taken as hypothesis-generating and not as definitive.

To our knowledge, only two other studies have used bulk RNA sequencing on AFX and PDS but never comparing the one against the other. A study from 2017 from Lai et al. [7] have compared eight AFXs with normal samples and fibroblast RNA sequencing data, and they found immune system-related, including tumor-associated macrophage (TAM) response (M2), EMT and GPCR ligand-binding-related pathways as the most significant differentially expressed genes. A second paper from 2020 from Klein et al. [8] compared 21 PDSs to six cutaneous squamous cell carcinoma samples, and they found PDGFRA and PDGFRB as notably upregulated in PDS as well as a fibroblast-related signature when compared to publicly available gene expression databases. There is also a more recent paper from 2024 from Klein et al. [9] where single-cell RNA sequencing of three AFXs and two PDSs revealed several other genes, including an increase in collagen subunit genes in PDS (COL4A1 and COL4A2), as well as enrichment in cell–matrix adhesion, EMT and vasculogenesis. While these three studies are difficult to compare with our study because of the different comparisons and strategies, some common themes, like receptor tyrosine kinase (RTK) signaling and immune-related pathways, appear to be particularly represented in these tumors and should probably be the logical focus of future larger studies.

While surgery remains the most common treatment for these tumors [12], the high TMB observed in both AFX and PDS [34] suggests that they may also be suitable candidates for immunotherapy, particularly in more advanced lesions. Although the latter is not yet a standard treatment for these tumors, it represents an active area of investigation, building on its success in other high-TMB cancers [35]. Importantly, studies like ours, focusing on gene expression profiling, can reveal novel molecular markers with potential diagnostic, prognostic, and therapeutic relevance. By elucidating the underlying biological mechanisms of these neoplasms—still largely unknown—our small work hopes to contribute to the identification of potential targeted strategies and align with the broader movement toward precision medicine.

Our study has some limitations that should be considered. First, the sample size of our cohort was relatively small, like other prior molecular studies [6,7,36,37,38], and validation of our findings in a larger cohort would help to confirm their robustness. Second, although we employed a broad panel of nearly 1400 genes, our analysis provides a mutational and gene expression profile limited to these targets; a comprehensive full transcriptome analysis could further expand and refine our observations.

## 5. Conclusions

In conclusion, our data show that AFX and PDS have the potential to be differentiated based on their gene expression profiles, demonstrating that, although they share a histologic spectrum and a common mutational background, they can be distinguishable at the molecular level, providing a rationale for their distinct biological behaviors.

## Figures and Tables

**Table 1:** Clinical and histopathologic features of our cohort.

	Age	Gender	Anat. Location	Ulceration	Subcutaneoous Invasion	Perineural Invasion	Intravascular Invasion	Necrosis	Mitoses	IHC	Treatment	F/U
AFX 01	80	M	L vertex scalp	NO	NO	NO	NO	NO	YES	Pos: CD10 (strong)—Neg: Soox10, p63, CK-AE1/3	Mohs micrographic surgery	No recurrence
AFX 02	72	M	L lower earlobe	NO	NO	NO	NO	NO	YES	Pos: CD10, CD68—Nneg: S100, Melan A, HM45, EMA, HMWCK, p63, CK 5/6, CK 8/18, CK AE1/3, desmin, calponin, ERG	Mohs micrographic surgery	No recurrence—deceased (for other causes)
AFX 03	84	M	Vertex scalp	YES	NO	NO	NO	NO	YES	Pos: CD10—Neg: S100, HMWCK, SMA	Mohs micrographic surgery	No recurrence—deceased (for other causes)
AFX 04	69	M	R parietal scalp	YES	NO	NO	NO	NO	YES	Pos: CD10—Neg: S100, HMWCK, desmin, CD31, ERG	Mohs micrographic surgery	No recurrence
AFX 05	91	F	Nose	NO	NO	NO	NO	NO	YES	Pos: Factor XIII (focally)—Neg: CD34, CK AE1/3, MNF116, CK Oscar, desmin, Melan A, S100	Re-excision	No recurrence—deceased (for other causes)
AFX 06	79	M	R dorsal hand	NO	NO	NO	NO	NO	YES	Neg: CK AE1/3, p63, S100	Mohs micrographic surgery	No recurrence
AFX 07	76	M	R parietal scalp	NO	NO	NO	NO	NO	YES	Pos: CD10—Neg: CK AE1/3, p63, S100	Mohs micrographic surgery	No recurrence—deceased (for other causes)
AFX 08	65	M	Scalp	NO	NO	NO	NO	NO	YES	Neg: CK AE1/3, p63, S100	Mohs micrographic surgery	No recurrence
AFX 09	89	F	R temple	NO	NO	NO	NO	NO	NO	Neg: S100, CK AE1/3, p63	Mohs micrographic surgery	No recurrence—deceased (for other causes)
AFX 10	85	M	L posterior scalp	NO	NO	NO	NO	NO	NO	Pos: CD68—Neg: S100, CK	Mohs micrographic surgery	No recurrence—deceased (for other causes)
PDS 01	42	M	Upper back/neck	YES	YES	NO	NO	YES	YES		Wide resection	Deceased (PDS)
PDS 02	69	M	L elbow	YES	YES	NO	NO	YES	YES		Wide resection + radiation	No recurrence
PDS 03	56	M	Face	YES	YES	YES	NO	YES	YES	Neg: CK AE1/3, CK Oscar, p40, HMWCK, EMA, desmin, S100, CD45, CD34, myogenin	Wide resection	Deceased (PDS)

## Data Availability

The data presented in this study are available on request from the corresponding author upon reasonable request.

## Supplementary Materials

The following supporting information can be downloaded at https://www.mdpi.com/article/10.3390/cancers18060934/s1, Figure S1: volcano plot of differentially genes overlapping with the literature in AFX and PDS; Figure S2: heatmaps for MAPK pathway, DNA repair and inflammatory response-related genes; Table S1: differential gene expression between AFX and PDS cases; Table S2: genes reported in the literature as either mutated or differential expressed in AFX and PDS; Table S3: results of over-representation analysis for Gene Ontology (GO) terms. Table S4: Results of the Gene Set Enrichment Analysis (GSEA).

## Abbreviations

The following abbreviations are used in this manuscript:

AD	Alternate allele depth
AFX	Atypical fibroxanthoma
cUPS	Cutaneous undifferentiated pleomorphic sarcoma
ECM	Extracellular matrix
EMT	Epithelial–mesenchymal transition
FDR	False discovery rate
FFPE	Formalin-fixed, paraffin-embedded
GO	Gene ontology
GOBP	Gene ontology biological process
GSEA	Gene set enrichment analysis
IRB	Institutional review board
MDSCs	Myeloid-derived suppressor cells
MMS	Mohs micrographic surgery
PCA	Principal component analysis
PDS	Pleomorphic dermal sarcoma
TMB	Tumor mutational burden
VAF	Variant allele frequency
VCFs	Variant call files
WLE	Wide local excision
