# Supplementary figures and images for: Gene Expression Profile (GEP) Comparison of Atypical Fibroxanthoma (AFX) and Pleomorphic Dermal Sarcoma (PDS)
Source: Cancers (Basel). 2026 Mar 13;18(6):934. doi: 10.3390/cancers18060934 (PMC13024464; doi:10.3390/cancers18060934)

# AFX vs PDS

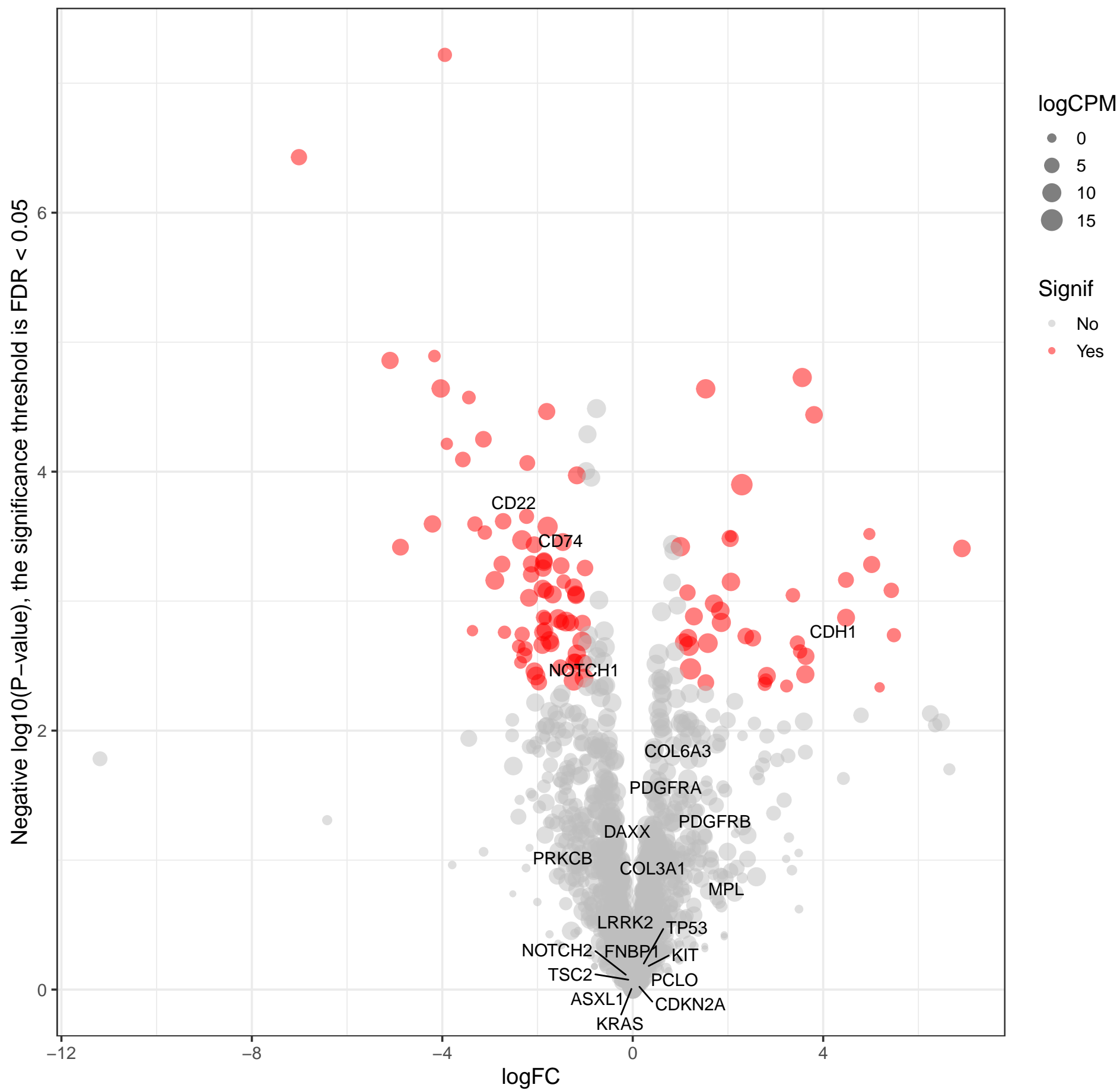

Supplement: Supplementary file 1 [file cancers-18-00934-s001.zip › Supplemental Figure S1 - LitGenes_VolcanoLabeled.pdf]

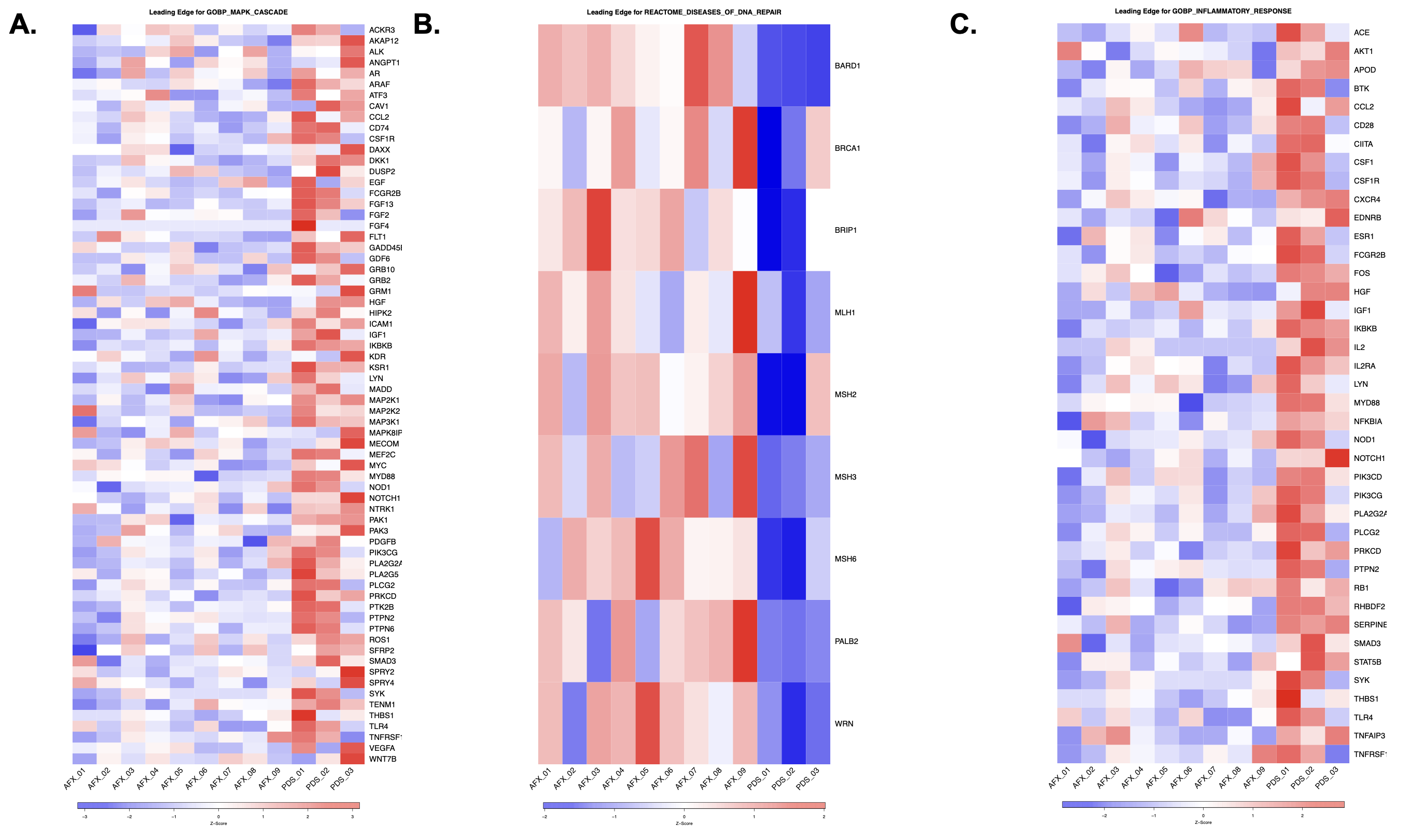

Supplement: Supplementary file 1 [file cancers-18-00934-s001.zip › Supplemental Figure S2 - Heatmaps.png]
